# 晚期肺癌患者肺功能指标与患者生存期的相关性研究

**DOI:** 10.3779/j.issn.1009-3419.2013.07.05

**Published:** 2013-07-20

**Authors:** 辉 葛, 正华 姜, 谦 黄, 慕云 朱, 捷 杨

**Affiliations:** 225001 扬州，扬州大学临床医学院，江苏省苏北人民医院呼吸内科 Department of Respiratory Medicine, Subei People's Hospital of Jiangsu Province, Yangzhou 225001, China

**Keywords:** 肺肿瘤, 肺功能, 生存期, Lung neoplasms, Lung function, Survival time

## Abstract

**背景与目的:**

晚期肺癌患者的治疗以提高疗效和改善生活质量为最终目的，肺功能指标是较好的评价指标。本研究探讨晚期肺癌患者肺功能改变及肺功能指标与患者生存期的相关性。

**方法:**

通过对59例晚期肺癌患者的肺功能进行检测，且与患者生存期进行相关性分析，并与63例健康人进行对照。

**结果:**

晚期肺癌患者的肺通气及弥散功能指标明显低于正常，与对照相比有统计学差异。肺功能指标中肺活量（vital capacity, VC）、第1秒用力呼出量（forced expiratory volume in one second, FEV_1_）、用力肺活量（gorced vital capacity, FVC）、最大呼气流速（peak expiratory flow, PEF）、最大呼气流速%（peak expiratory flow%, PEF%）、最大通气量（maximal ventilatory volume, MVV）与患者生存期呈正相关（*r*分别为0.29、0.28、0.28、0.27、0.26、0.28，*P* < 0.05），残气量/肺总量（residual volume/total lung, RV/TLC）值与患者生存期呈负相关（*r*=-0.31, *P* < 0.05）。

**结论:**

肺癌患者存在肺功能的减退，肺癌患者肺功能指标中VC、FEV_1_、FVC、PEF、PEF%、MVV、RV/TCL值与患者生存期具有相关性，肺功能的部分指标可作为肺癌患者预后评估的重要因素之一。

## Abstract (en)

**Background and objective:**

To those patients with advanced lung cancer, the ultimate objective is to improve the curative effect and quality of life, lung function indexes are an important factor. We investigate the change of lung function and the relationship between pulmonary function indexs and survival time in patients with advanced lung cancer.

**Methods:**

Lung function was detected in 59 cases with lung cancer and 63 normal controls. The relationship between pulmonary function indexs and survival time was analyzed.

**Results:**

There was significant difference in ventilation function and diffusing capacity between in lung cancer group and control group. Vital capacity (VC), forced expiratory volume in one second (FEV_1_), forced vital capacity (FVC), peak expiratory flow (PEF), peak expiratory flow% (PEF%), maximal ventilatory volume (MVV) were positively correlated with survival time in patients with advanced lung cancer (*r*=0.29, 0.28, 0.28, 0.27, 0.26, 0.28, *P* < 0.05), residual volume/total lung capacity was negatively correlated with survival time (*r*=-0.31, *P* < 0.05).

**Conclusion:**

The lung function decreases in the patients with lung cancer. VC, FEV_1_, FVC, PEF, PEF%, MVV, residual volume/total lung capacity were correlated with survival time in patients with advanced lung cancer. The pulmonary function indexs were important marker of prognosis in patients with lung cancer.

肺癌是呼吸系统的常见肿瘤，其发病率及死亡率呈逐年上升，资料显示肺癌已成为城市男性恶性肿瘤死因首位。大多数就诊患者已诊断为晚期肺癌，无手术指征。对于不能手术的肺癌患者，提高疗效和改善生活质量是最终目的，后者近些年来越来越受到关注。本研究通过对59例晚期肺癌患者的肺功能进行检测，并与63例正常人进行对照，同时将肺功能指标与肺癌患者生存期进行相关性研究，以了解肺癌患者的肺功能改变，为肺癌临床病情发展及预后的评估提供参考。

## 1 对象与方法

### 1.1 研究对象

选择2009年1月-2011年10月在我院住院患者，59例肺癌患者均经纤维支气管镜活检、CT引导下肺穿刺活检后病理证实为原发性肺癌，男性51例，女性8例，年龄40岁-77岁，平均年龄（63.68±8.98）岁，腺癌25例，腺鳞癌1例，鳞癌19例，小细胞肺癌14例，所有患者均行胸部CT、头颅MRI、B超、ECT等检查，根据TNM分期，均为晚期肺癌。正常对照组：63例均为健康体检者，其中男性51例，女性12例，年龄41岁-88岁，平均年龄（56.76±8.90）岁。

### 1.2 方法

所有患者治疗前及健康体检者均采用德国耶格肺功能仪进行肺功能检测。测定前输入患者的身高、体重、性别、年龄、温度、大气压等指标，由电脑自动产生相应的预测值。对所有患者进行肺活量（vital capacity, VC）、第1秒用力呼出量（forced expiratory volume in one second, FEV_1_）、用力肺活量（forced vital capacity, FVC）、第1秒用力呼出量/用力肺活量（FEV_1_/FVC）、最大呼气流速（peak expiratory flow, PEF）、最大通气量（maximal ventilatory volume, MVV）、残气量/肺总量（RV/TLC）及一氧化碳弥散量（diffusing capacity of carbon monoxide, DLCO）、弥散系数（DLCO/VA）检测，采用实测值占预测值百分比来表示结果。

### 1.3 统计学处理

采用PEMS 3.1软件对数据进行处理，所有结果均采用Mean±SD表示，两样本均数的比较采用*t*检测。肺功能指标与生存期关系用直线相关分析。以*P* < 0.05为差异具有统计学意义。

## 2 结果

### 2.1 肺癌患者与正常对照组肺功能指标的比较

肺癌组患者与正常对照组相比，肺通气功能的RV指标高于正常对照组，但统计学上无明显差异; RV/TCL指标高于对照组，其余各项指标均低于正常对照组，有明显统计学差异。肺癌组患者与正常对照组相比，肺癌组小气道功能指标V25、V25%、V50、V50%分别为4.29±1.96、65.45±29.82、2.03±1.18、50.76±27.26，均明显低于正常对照组，有明显统计学差异。肺癌组与正常对照组相比，肺弥散功能的各项指标分别为5.57±1.86、68.21±22.09、1.26±0.36、91.09±22.42均明显低于正常对照组，有明显统计学差异（表 1）。

**1:** 肺癌组及正常对照组肺功能检测结果分析 Lung function test results of lung cancer and normal group

Lung functional indexes	Control (*n*=63)	Lung cancer (*n*=59)	*t*	*P*
VC	3.74±0.73	2.93±0.72	6.179, 9	< 0.001
VC%	103.24±14.85	82.29±17.86	7.062, 9	< 0.001
FEV_1_	3.27±0.68	2.12±0.65	9.527, 9	< 0.001
FEV_1_%	112.66±14.52	77.03±20.73	11.052, 4	< 0.001
FVC	3.64±0.74	2.90±0.73	5.534, 5	< 0.001
FVC%	101.51±18.42	83.68±17.99	5.401, 8	< 0.001
FEV_1_/FVC%	90.02±4.57	72.73±11.44	11.092, 2	< 0.001
PEF	7.81±1.87	5.73±1.80	6.196, 7	< 0.001
PEF%	104.64±22.59	77.51±22.80	6.600, 7	< 0.001
MVV	117.32±30.67	69.82±20.48	9.988, 3	< 0.001
MVV%	108.99±25.84	65.47±17.96	10.731, 8	< 0.001
RV	1.99±0.44	2.29±3.53	0.665, 7	> 0.05
RV/TCL	35.26±7.22	40.20±9.53	3.243, 3	< 0.01
V25	7.27±1.88	4.29±1.96	8.582, 0	< 0.001
V25%	110.64±25.66	65.45±29.82	8.989, 3	< 0.001
V50	4.43±1.56	2.03±1.18	9.530, 1	< 0.001
V50%	104.35±35.56	50.76±27.26	9.295, 7	< 0.001
DLCO	8.42±1.85	5.57±1.86	8.474, 1	< 0.001
DLCO%	97.25±13.59	68.21±22.09	8.808, 2	< 0.001
DLCO/VA	1.51±0.2	1.26±0.36	4.917, 9	< 0.001
DLCO/VA%	101.48±13.72	91.09±22.42	3.109, 2	< 0.01
VC: vital capacity; FEV_1_: forced expiratory volume in one second; FVC: forced vital capacity; PEF: peak expiratory flow; MVV: maximal ventilatory volume; DLCO: diffusing capacity of carbon monoxide.				

### 2.2 肺癌患者肺功能指标与患者生存期的相关性

如表 2所示，肺癌组59例患者均获随访，随访率100%。生存期为确诊日至患者死亡或末次随访的时间（2012年1月23日）。随访肺癌患者生存期为122天-936天，中位生存期为357天。肺功能指标中VC、FEV_1_、FVC、PEF、PEF%、MVV与患者生存期呈正相关（*r*分别为0.29、0.28、0.28、0.27、0.26、0.28，*P* < 0.05），RV/TCL值与患者生存期呈负相关（*r*=-0.31, *P* < 0.05）。

**2:** 肺癌患者肺功能指标与患者生存期的相关系数 The coefficient correlation of lung function indexes and survival time of patients with lung cancer

Lung functional index	Coefficient correlation	*t*	*P*
VC	0.29	2.246, 7	< 0.05
VC%	0.21	1.647, 9	> 0.05
FEV_1_	0.28	2.206, 6	< 0.05
FEV_1_%	0.22	1.712, 4	> 0.05
FVC	0.28	2.226, 8	< 0.05
FVC%	0.22	1.701, 0	> 0.05
FEV_1_/FVC%	0.10	0.766, 1	> 0.05
PEF	0.27	2.109, 3	< 0.05
PEF%	0.26	2.061, 0	< 0.05
V25	0.16	1.258, 8	> 0.05
V25%	0.16	1.235, 0	> 0.05
V50	0.18	1.417, 7	> 0.05
V50%	0.17	1.317, 4	> 0.05
MVV	0.28	2.237, 0	< 0.05
MVV%	0.24	1.877, 5	> 0.05
RV	0.09	0.680, 1	> 0.05
RV/TCL	-0.31	-2.440, 7	< 0.05
DLCO	0.21	1.582, 6	> 0.05
DLCO%	0.12	0.939, 1	> 0.05
DLCO/VA	0.19	1.424, 6	> 0.05
DLCO/VA%	0.14	1.046, 9	> 0.05

## 3 讨论

肺癌是呼吸系统恶性肿瘤，发病率呈逐年上升趋势，治愈率低，肺癌的5年生存率仅为20%左右，其原因主要是因为新发病例中，75%属于晚期肺癌。肺癌是恶性肿瘤中死亡率最高的肿瘤之一，每年全世界约有100多万新发病例产生，其中超过1/3病例在诊断时已有远处转移，仅有15%肺癌患者诊断为早期^[1]^。

肺功能可能在对肺癌患者的筛查中扮演一种有用的角色，研究^[2]^表明阻塞性肺疾病与肺癌有一定相关性，近来一些研究^[3-5]^显示50%-80%确诊为肺癌患者中存在有慢性阻塞性肺疾病，另也有研究^[6]^表明阻塞性和限制性肺功能损害与肺癌发病率增高密切相关。周怡等^[7]^研究，对肺癌患者进行肺功能检测，发现肺癌患者FVC、FEV_1_、PEF、V25、V50的下降，表明肺癌患者存在阻塞性通气功能障碍。本研究结果表明肺癌组患者与正常对照组相比，肺通气功能的RV指标高于正常对照组，但统计学上无差异; RV/TCL指标高于对照组，其余各项指标均低于正常对照组，统计学上有明显差异。肺癌组患者与正常对照组相比，肺癌组小气道功能指标V25、V25%、V50、V50%分别为4.29±1.96、65.45±29.82、2.03±1.18、50.76±27.26，均明显低于正常对照组，统计学上有显著差异。这些研究均表明肺癌患者合并阻塞性通气功能障碍。肺癌患者通气功能障碍产生的机制如下：①肺癌患者多有长期吸烟史，部分患者多伴有慢性阻塞性肺疾病和肺气肿，从而引起阻塞性通气功能障碍; ②肺癌本身由于肺部肿块、肺不张及阻塞性肺炎等因素，导致可以通气的肺组织减少，从而引起阻塞性通气功能障碍; ③肺癌患者由于肿瘤因素，前列腺素分泌增多，引起细支气管痉挛或部分肺泡闭合，进而引起阻塞性通气功能障碍。

弥散功能是肺换气功能的重要组成部分，通过弥散O_2_从肺泡进入到肺毛细血管，而CO_2_则从肺毛细血管排出到肺泡。正常人的弥散功能 > 预测值的80%。Margaritora等^[8, 9]^研究证实DLCO是一种有效的肺部并发症的预测因素。Baser等^[10]^的研究表明术前低水平的DLCO（< 45%）与术后肺部并发症的发病率与死亡率增加是相关的，且预示较差的生活质量。本研究结果表明肺癌组与正常对照组相比，肺弥散功能的各项指标分别为5.59±1.94、65.21±26.55、1.28±0.38、87.62±31.41均明显低于正常对照组，有统计学差异。这说明肺癌导致患者弥散功能下降。其主要原因如下：①肿瘤压迫阻塞气管引起阻塞性炎症、肺不张及胸膜病变等原因，引起肺容量减少、通气功能受限，造成弥散面积减少; 同时周围肺组织的代偿使通气量增加，引起通气/血流比例失调; ②癌肿压迫血管，血流受阻，血流量减少，通气血流比例增高; ③肺癌细胞还可经血流转移，阻塞小血管，导致通气血流比例的进一步失调; ④肺癌患者大多为中老年者，大多有长期吸烟史，有的患者伴随有慢性肺部疾病。

虽然众所周知，对于考虑手术的非小细胞肺癌患者，肺功能是一种重要的预后因素^[11, 12]^，而对于晚期非小细胞肺癌患者预后评估方面肺功能并没有被应用。本研究结果显示肺癌患者肺功能指标中VC、FEV_1_、FVC、PEF、PEF%、MVV与患者生存期呈正相关（*r*分别为0.29、0.28、0.28、0.27、0.26、0.28，*P* < 0.05），RV/TCL值与患者生存期呈负相关（*r*=-0.31, *P* < 0.05）。FEV_1_是第1秒钟呼出的容积，根据慢性阻塞性肺疾病全球指南FEV_1_是作为气道阻塞的判定标准，也是慢性阻塞性肺疾病患者死亡率判定的重要预测因子^[13, 14]^。有研究^[15]^表明FEV_1_的下降是肺癌的一种危险因素，减低的FEV_1_与中晚期非小细胞肺癌死亡率密切相关，提示FEV_1_是预后判断的重要因素之一，Calabro等^[2]^研究也显示肺癌患者的肺功能指标FEV_1_明显低于正常对照组，降低的FEV_1_是预测肺癌的高危因素之一。我们的研究表明FEV_1_与肺癌患者的生存期呈正相关，也就是说低的FEV_1_预示患者较短的生存期，这与上述的研究是一致的。肺活量是指最大吸气后能呼出的最大气量，由深吸气量与补呼气容积组成。肺活量减低见于胸廓﹑肺扩张受限，肺组织损害，气道阻塞。最大通气量是指单位时间内以尽快的速度和尽可能深的幅度进行呼吸所得到的通气量。一般嘱患者深快呼吸12 s，将得到的通气量乘以5即为每分钟的最大通气量。它是一项简单的负荷试验，用以衡量气道的通畅度﹑肺和胸廓的弹性和呼吸肌的力量。通常用作能否进行胸科手术的指标。用力肺活量是指用最快的速度所作的呼气肺活量。并可由此计算出第1秒钟呼出的容积和第1秒钟呼出容积占用力肺活量之比。用力肺活量是当前最佳的测定项目，可以反映较大气道的呼气期阻力。可用作慢性支气管炎﹑支气管哮喘和肺气肿的辅助诊断手段，也可考核支气管扩张剂的疗效。呼气高峰流量是在肺总量位时，猛力快速吹向最高呼气流量计，观察最高呼气流速。残气/肺总量是表明肺气肿的指标，表明阻塞性通气功能严重程度的一项指标。我们的研究还显示VC、FVC、PEF、PEF%、MVV与肺癌患者生存期呈正相关，RV/TCL值与患者生存期呈负相关（*r*=-0.31, *P* < 0.05），也就是说明VC、FVC、PEF、PEF%、MVV的下降预示患者短的生存期，增高的RV/TCL也预示患者较短的生存期，这方面的研究报道较少。

综上所述，肺癌患者肺通气功能与弥散功能指标明显下降，肺癌患者肺功能指标中VC、FEV_1_、FVC、PEF、PEF%、MVV与患者生存期呈正相关，RV/TCL值与患者生存期呈负相关，肺功能的部分指标可作为肺癌患者预后评估的重要因素之一。
